# Application of antagonist *Bacillus amyloliquefaciens* NCPSJ7 against *Botrytis cinerea* in postharvest Red Globe grapes

**DOI:** 10.1002/fsn3.1434

**Published:** 2020-02-07

**Authors:** Qingxin Zhou, Maorun Fu, Minhui Xu, Xiangyan Chen, Jiying Qiu, Fengli Wang, Ran Yan, Junhua Wang, Shuangzhi Zhao, Xue Xin, Leilei Chen

**Affiliations:** ^1^ Institute of Agro‐Food Science and Technology Shandong Academy of Agricultural Sciences/Key Laboratory of Agro‐Products Processing Technology of Shandong Province/Key Laboratory of Novel Food Resources Processing Ministry of Agriculture Jinan China; ^2^ College of Life Science Shandong Normal University Jinan China; ^3^ College of Food Science and Engineering Qilu University of Technology (Shandong Academy of Sciences) Jinan China

**Keywords:** antioxidant capacity, *Bacillus amyloliquefaciens* NCPSJ7, *Botrytis cinerea*, Red Globe grape, resistance‐related enzymes

## Abstract

We investigated the effects and possible mechanisms of *Bacillus amyloliquefaciens* NCPSJ7 against the gray mold caused by *Botrytis cinerea* in the postharvest Red Globe grapes. The disease incidence, lesion diameter, decay index, and some resistance‐related enzymes were evaluated. The antioxidant capacity of grape treated with 1 × 10^4^ CFU/ml *B. cinerea* alone and combined with 1 × 10^7^ CFU/ml NCPSJ7 was also determined. The results showed that NCPSJ7 + *B. cinerea* reduced the disease incidence, lesion diameter, and decay index of postharvest grapes and enhanced the activities of polyphenol oxidase, peroxidase, chitinase, and β‐1,3‐glucanase during different storage periods. Furthermore, the oxidative resistance, demonstrated by an escalating trend in the total phenolic content, DPPH free radical clearance rate, reducing power, and superoxide anion clearance rate after lesion presence, was improved. However, NCPSJ7 showed an inhibitory effect on gray mold, but resulted in the reduced antioxidant capacity in the grapes.

## Graphical abstract

*Bacillus amyloliquefaciens* NCPSJ7 reduced the disease incidence, lesion diameter, and decay index of postharvest Red Globe grapes. About 107 CFU/ml of *B. amyloliquefaciens* NCPSJ7 is effective in inducing resistance against gray mold in Red Globe grapes.

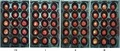

## 1 INTRODUCTION

The decay caused by microbial invasion results in major losses of postharvest fruits and vegetables, which creates a huge economic burden. It has been estimated that about 20%–25% of harvested fruits and vegetables decay from filamentous fungal infections during postharvest handling, even in developed countries; therefore, the control of such infections is critical (Calvo, Marco, Blanco, Oria, & Venturini, 2017). Gray mold, caused by *Botrytis cinerea*, is one of the most destructive diseases in grape production (Fu, Qu, Yang, & Zhang, 2016; Jaspers, Seyb, Trought, & Balasubramaniam, 2016; Liu et al., 2016). At present, the main prevention and control methods for grape gray mold include the use of chemical fungicides, such as cyprodinil (Latorre & Torres, 2012), pyrimethanil (Gil, Becker, & Viegas, 2014), fludioxonil (Zhang et al., 2015), and fenhexamid (Petit et al., 2010). However, the irrational use and inherent weaknesses of these chemical drugs have resulted in serious environmental pollution (Li et al., 2016), fungicide resistance (De Miccolis Angelini et al., 2014), and pesticide residues (Cabras et al., 1997), which have affected the ecosystem balance and human health (Esteve‐Turrillas, Agulló, Abad‐Somovilla, Mercader, & Abad‐Fuentes, 2016; Orton, Rosivatz, Scholze, & Kortenkamp, 2011).

Biocontrol plays an important role in the postharvest preservation of vegetables and fruits. The method has many merits, such as its safety, efficiency (Han et al., 2016), and lack of both pollution and ecological disturbance (Droby, Wisniewski, Teixidó, & Jijaklie, 2016). Biological control using microorganisms, such as *Bacillus* spp., *Pseudomonas* spp., *Trichoderma* spp., and some plant growth‐promoting rhizobacterial strains, has been proposed for the control of diseases caused by soilborne pathogens (Sotoyama, Akutsu, & Nakajima, 2016). *Bacillus amyloliquefaciens*, a free‐living soil bacterium, has obvious antagonistic effects through the secretion of enzymes (chitinase, β‐1,3‐glucanase) (Arrebola, Sivakumar, & Korsten, 2010), lipopeptides (surfactin, iturin, and fengycin) (Hanif et al., 2019; Ongena & Jacques, 2008), volatile compounds (bacillaene, bacilysin, and difficidin) (Chen, Koumoutsi, Scholz, & Borriss, 2009; Wu, Zhou, Li, & Ma, 2019), and other antifungal substances (Scholz et al., 2011; Wang, Wu, Chen, Lin, & Yang, 2016).

In recent years, *B. amyloliquefaciens* application on the decay of postharvest fruits and vegetables caused by pathogenic microorganisms has become a hot topic locally and abroad (Calvo, Mendiara, Arias, Blanco, & Venturini, 2019; Chen, Tian, Luo, Cheng, & Long, 2018; Li et al., 2013; Nam et al., 2015). However, few published reports have investigated the effects of *B. amyloliquefaciens* NCPSJ7 on inhibiting the growth of gray mold in grapes. Thus, the objectives of this study were to investigate resistance‐related enzymes and the antioxidant capacity of Red Globe grapes treated with *Botrytis cinerea* alone and a combination of *Botrytis cinerea* and *Bacillus amyloliquefaciens* NCPSJ7 in order to determine the possible biocontrol mechanisms involved. Our results could provide a useful biocontrol technology for storing and preserving postharvest fruits and vegetables.

## 2 MATERIALS AND METHODS

### 2.1 Fruits

The Red Globe grapes used in this study were collected from the grape research base (Jinan Institute of Agricultural Sciences). The fruits were brought back to the laboratory within 1 hr and processed by washing with 0.5% NaClO solution and rinsing with distilled water. Following this, the grapes were left to dry at 25°C and then used in the inoculation experiments.

### 2.2 Pathogens

*Botrytis cinerea*, which was isolated from grapes, was obtained from the Institute of Botany, Chinese Academy of Sciences (CMCC 3.3790). It was maintained on potato dextrose agar (PDA) medium and stored at 4°C. The PDA medium contained 200 g/L potato extract, 20 g/L dextrose, and 20 g/L agar (Aoboxing Bio‐Technology Co.). The pathogen inoculums were aqueous conidial suspensions prepared from 7‐day‐old dish cultures incubated at 25°C. The cultures were flooded with sterile deionized water (diH_2_O) containing 0.01% Tween 80 solution, and the suspension was passed through two layers of sterile cheesecloth to remove hyphal fragments. The conidial concentration of the pathogen was adjusted to 1 × 10^4^ spores/ml using a hemocytometer.

### 2.3 Strains

Antagonistic bacterium *B. amyloliquefaciens* NCPSJ7 was obtained from the Institute of Agri‐food, Shandong Academy of Agricultural Sciences. It was maintained in glycerin tubes and stored at −20°C. A 0.5 ml aliquot of cells from the glycerin tube was inoculated into a 500‐ml flask containing 100 ml of nutrient broth (NB) medium (3 g/L beef extract, 10 g/L peptone, and 5 g/L NaCl; pH 7.2–7.4). The flask was incubated on a rotary shaker at 150 r/min for 18 hr at 33°C. Then, 1 ml of the cultivated bacterial suspension was added to a new shake flask containing 100 ml of NB medium and incubated under the same conditions, as described above. The test required different concentrations of NCPSJ7, which were determined by viable counts on solid plates and adjusted with sterile saline with a 0.01% Tween 80 solution.

### 2.4 Effects of NCPSJ7 on the disease incidence, lesion diameter, and decay index in infected Red Globe grapes

The fruits were prepared for inoculation by a wound (3 mm deep × 3 mm wide) at the equator in each fruit using a sterile needle. The experiment was divided into four treatment groups: CK (20 μl of sterile distilled water without any inoculation); I (20 μl of 1 × 10^6^ NCPSJ7 spores/ml); II (20 μl of 1 × 10^7^ NCPSJ7 spores/ml); and III (20 μl of 1 × 10^8^ NCPSJ7 spores/ml). Each treatment was injected into each wound with a pipette, respectively. Then, after air‐drying the fruits for about 12 hr, 10 μl of 1 × 10^4^ *B. cinerea* spores/ml was inoculated into each wound. The treated grapes were arranged in packing boxes (280 mm × 180 mm × 40 mm) and then covered with a polyethylene bag (350 mm × 250 mm, thickness 0.02 mm) and stored at 25°C. The disease incidence from 3 to 5 days was calculated, and the decay index (DI) and lesion diameter were evaluated at the end of storage (5 days). There were three replicates of each treatment, and each replicate included 15 fruits. The disease severity of a single grape was assessed according to the following lesion area: level 0 (0% lesion area on fruit surface); level 1 (25% lesion area on fruit surface); level 2 (50% lesion area on fruit surface); level 3 (75% lesion area on fruit surface); and level 4 (100% lesion area on fruit surface). The DI was calculated using the formula established by Zhang and Fu (2018): DI = *df*/ND, where d is the level of decay severity on the grape, f is the quantity of grapes in this level, N is the total number of grapes examined, and D is the highest degree of disease severity measured on a severity scale.

### 2.5 Effects of NCPSJ7 on resistance‐related enzymes in infected Red Globe grapes

The experiment was divided into three treatment groups, as follows: (a) (CK group: 20 μl of sterile distilled water with any inoculation); (b) (*B. cinerea* group: 20 μl of sterile distilled water was pipetted into each wound, and after air‐drying the fruit for about 12 hr, 10 μl of 1 × 10^4^ *B. cinerea* spores/ml was inoculated into each wound as well); and (c) (NCPSJ7 + *B. cinerea* group: 20 μl of 1 × 10^7^ NCPSJ7 spores/ml was injected into each wound with a pipette, and after air‐drying the fruit for about 12 hr, 10 μl of 1 × 10^4^ *B. cinerea* spores/ml was also inoculated into each wound). The treated fruits were arranged in packing boxes (280 mm × 180 mm × 40 mm) that were then covered with a polyethylene bag (350 mm × 250 mm, thickness 0.02 mm) and stored at 25°C. Each treatment consisted of six packing boxes, with 15 fruits per box. One box was taken from the different treatment groups every day for testing of the antioxidant capacity and enzyme activities in the grapes. For groups A and B, 1 cm of skin was taken around the wound in grapes, whereas 1 cm of skin was taken at the location of the lesion and the intact tissue for group C. The experiment was repeated twice.

### 2.6 Determination of polyphenol oxidase (PPO) and peroxidase (POD) enzyme activities

Skin samples (~2 g) from 15 grapes in each treatment group were crushed in 10 ml of 50 mM sodium phosphate buffer (pH 6.5) or 7 ml of 100 mM sodium phosphate buffer (pH 6.0) in an ice bath with a mortar and pestle. Then, the crushed skin was centrifuged at 8,500 *g*/min for 10 min at 4°C and the supernatant was used for the enzyme assay.

The activity of PPO (EC 1.14.18.1) was determined according to previously described methods, with minor modifications (Serradell et al., 2000). The change in absorbance at 525 nm was monitored for 10 min. The specific activity was expressed as U/g fresh weight (FW), where one unit (U) was defined as an absorbance increase of 0.01.

The POD enzyme (EC 1.11.1.7) activity was determined according to the method reported by Hammerschmidt, Nuckles, and Kuć (1982), with suitable modifications. The increase in absorbance at 460 nm was monitored every 30 s for 180 s. The specific activity was expressed as U/g FW, where one U was defined as an absorbance increase of 1.

### 2.7 Determination of chitinase and glucanase activity

Skin samples (2 g) obtained from 15 grapes for each treatment group were extracted in 7 ml of 0.1 mol/L NaAc‐HAc buffer solution (pH 5.2), which consisted of 1 mmol/L ethylenediaminetetraacetic acid (EDTA), 5 mmol/L β‐mercaptoethanol, and 1 g/L ascorbic acid and was ground at 4°C. The extracts were centrifuged at 8,500 *g*/min for 15 min at 4°C, and the supernatant was used for the chitinase (CHI) and β‐1,3 glucanase (GLU) assays.

The CHI enzyme (EC 3.2.1.14) activity was assessed by taking an aliquot (1.0 ml) of the crude enzyme preparation and mixing it with 1.0 ml of 1% colloidal chitin (Sigma) and 1.0 ml of 100 mmol/L NaAc‐HAc buffer solution (pH 5.2) and incubating it in a water bath at 37°C for 1 hr. The reaction mixture was then centrifuged at 8,500 *g*/min for 15 min at 4°C. The supernatants were used according to the method of Gao, Qi, Xie, Huang, and Lin (2017), with minor modifications. The reaction in 0.4 ml of the supernatant was stopped by adding 0.4 ml of 3,5‐dinitrosalicylic acid (DNS) reagent and boiling the mixture for 5 min. The mixture was then diluted to 25 ml. The absorbance at 540 nm was spectrophotometrically determined and estimated by a standard curve of *N*‐acetyl‐d‐glucosamine (NAG) and expressed as U/g FW. The amount of enzyme producing 1 × 10^–9^ mol NAG/second under these assay conditions was defined as one unit. Each value is presented as the mean of three replicate assays.

The GLU enzyme (EC 3.2.1.73) activity was detected using 3,5‐dinitrosalicylic acid (DNS) reagent and determined at 540 nm (Nelson, 1944). The GLU enzyme activity was calculated according to a standard curve of glucose and expressed as U/g FW. The amount of enzyme producing 1 × 10^–9^ mol glucose per second under these assay conditions was defined as one unit. Each value is presented as the mean of three replicate assays.

### 2.8 Effects of NCPSJ7 on the antioxidant capacity in infected Red Globe grapes

Skin samples (1 g) from 15 grapes for each treatment group were extracted with 20 ml of a 70% (v/v) aqueous ethanol solution under a sonicating bath for 30 min at 25°C. The extract solution was centrifuged at 8,500 *g*/min for 10 min at 4°C. The antioxidant capacity of the solution was immediately determined.

The total phenolic content (TPC) was measured using the Folin–Ciocalteu method (Fu et al., 2016) and detected at a wavelength of 760 nm using a spectrophotometer. The TPC was determined as micrograms of gallic acid equivalents per milliliter of extract solution. The equation of the calibration curve was y = 0.0585 × −0.0176, with a correlation coefficient of *R* ^2^ = .9969.

The DPPH free radical scavenging activity was determined according to the method of Qu et al. (2016) and measured at a wavelength of 517 nm. The DPPH free radical scavenging activity was calculated using the following equation: scavenging activity (%) = [1 – (A_1_/A_0_)]/100, where A_0_ is the absorbance of the blank, and A_1_ is the absorbance in the presence of the extract.

The reducing power was determined according to the method of Qu et al. (2016), with minor modifications. In brief, 1.0 ml of the working extract solution was mixed with 2.5 ml of 0.2 mol/L phosphate buffer (pH 6.6) and 2.5 ml of 0.03 mol/L potassium ferricyanide. An aliquot (2.5 ml) of 0.6 mol/L trichloroacetic acid was added to the mixture, which was then centrifuged at 8,500 *g*/min for 10 min. The upper layer of the solution (2.5 ml) was mixed with 2.5 ml of distilled water and 0.5 ml of 0.006 mol/L FeCl_3_, and the absorbance was measured at a wavelength of 700 nm using a spectrophotometer.

The superoxide anion scavenging activity was measured using the previously described xanthine/xanthine oxidase method (Fu, He, Zhao, Yang, & Mao, 2009).

### 2.9 Statistical analyses

The data were statistically analyzed using the Statistical Package for Social Sciences version 15.0 (SPSS Inc.) and MS Office Excel 2003. One‐way ANOVA was performed to evaluate the difference between the groups, followed by Duncan's test for multiple comparisons. *p *< .05 was considered statistically significant.

## 3 RESULTS

### 3.1 Effects of NCPSJ7 on the disease incidence, lesion diameter, and decay index in infected Red Globe grapes

**Figure 1:**
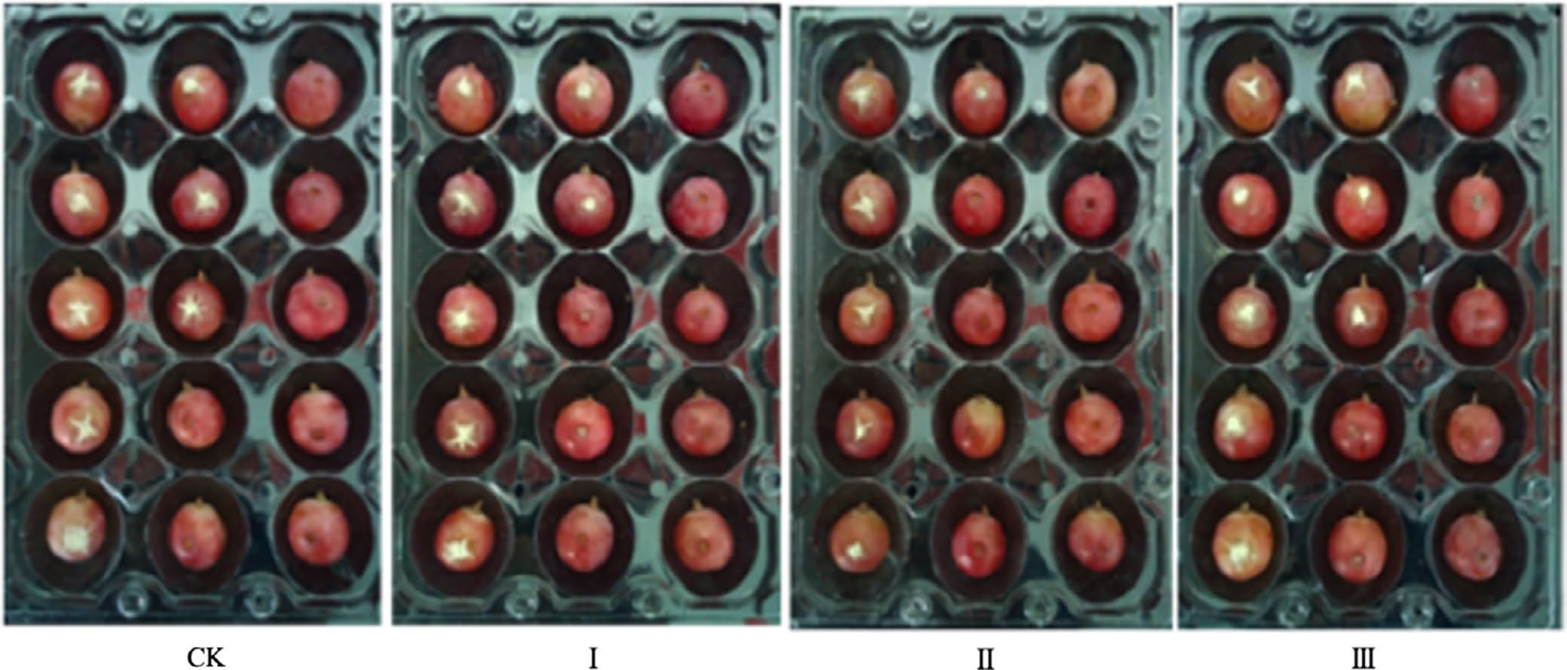
The inhibitory effect of NCPSJ7 treatments with different concentration of cells against *Botrytis cinerea* on grapes. (CK, control; I, 10^6^ CFU/ml; II, 10^7^ CFU/ml; and III, 10^8^ CFU/ml)

Figure 1 shows the overall appearance of grapes treated by 1 × 10^4^ CFU/ml *B. cinerea* and different concentrations of NCPSJ7. From the numbers and areas of infected grapes, 10^7^ CFU/ml of NCPSJ7 inhibited the growth of *B. cinerea* better than CK and other treatments.

**Figure 2:**
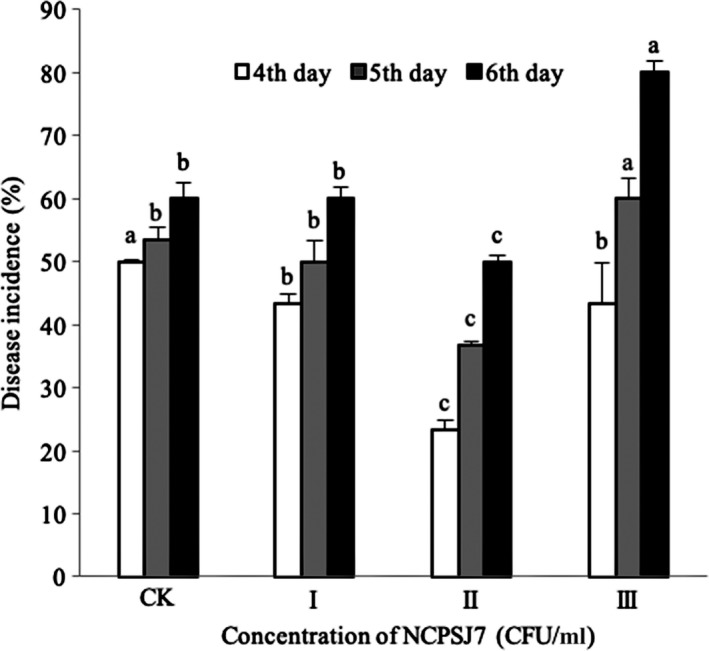
The inhibitory action of different concentrations of NCPSJ7 on disease incidence against 1 × 10^4^ CFU/ml of *Botrytis cinerea* (CK, control; I, 10^6^ CFU/ml; II, 10^7^ CFU/ml; and III, 10^8^ CFU/ml). Values are means ± standard errors (*n* = 3). Different lowercase letters indicate the significant difference between treatments (*p* < .05)

The graph of the disease incidence is shown in Figure 2. The group treated with 10^7^ CFU/ml of NCPSJ7 had a significantly lower disease incidence (23.3% and 35.6% on the 4th and 5th days, respectively) than the CK group (50% and 51% on the 4th and 5th days, respectively) and other treatment groups. The 10^6^ CFU/ml treatment group had a similar incidence to the control, whereas the 10^8^ CFU/ml treatment group showed the highest disease incidence. That is, the order of disease incidence was II < CK <I < III (*p* < .05). This may be due to the higher concentrations of *B. amyloliquefaciens* strain NCPSJ7 requiring more nutrients from the grapes, and, thereby, leading to greater damage to the fruit. Following pathogen inoculation, the antagonistic bacteria not only failed to reduce the disease incidence but instead increased it. From the results, the 10^7^ CFU/ml treatment group was considered to be more appropriate for fruit storage.

**Figure 3:**
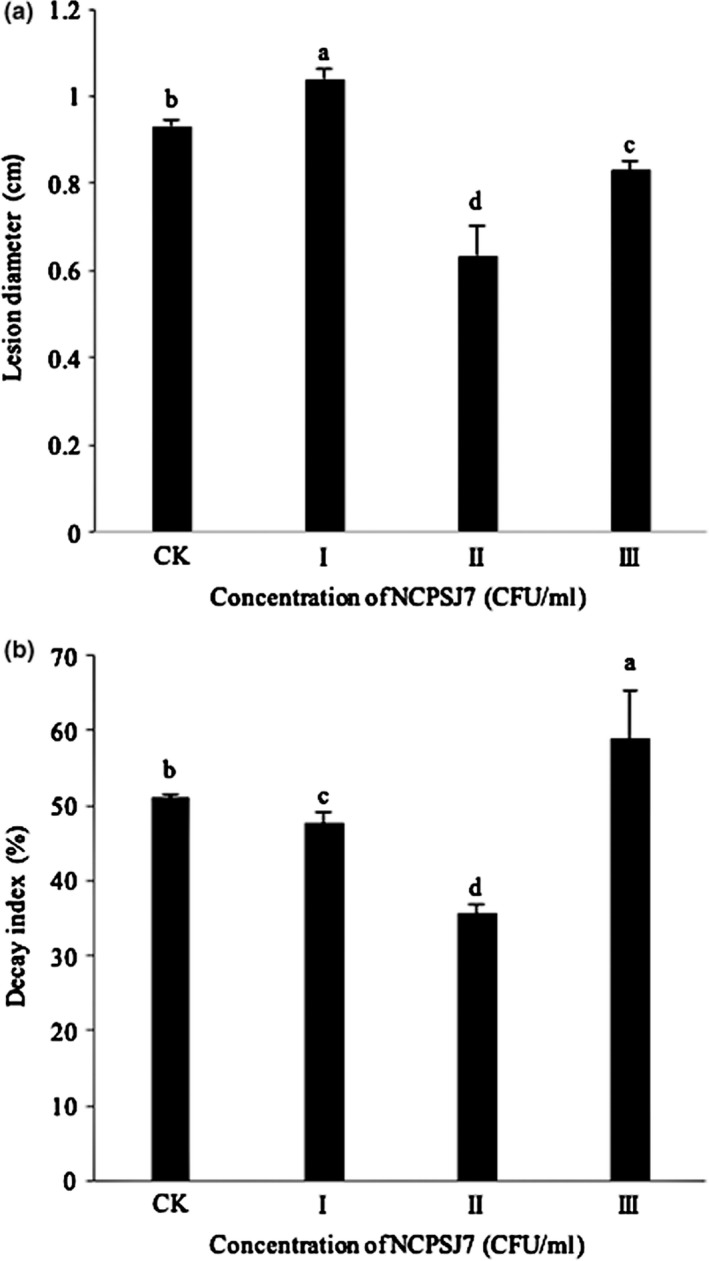
The inhibitory action of different concentrations of NCPSJ7 on lesion diameter and decay index against 1 × 10^4^CFU/ml of *Botrytis cinerea* (CK, control; I, 10^6^ CFU/ml; II, 10^7^ CFU/ml; and III, 10^8^ CFU/ml). Values are means ± standard errors (*n* = 3). Different lowercase letters indicate the significant difference between treatments (*p* < .05)

As seen from the graphs of lesion diameter in Figure 3a, the 10^7^ and 10^8^ CFU/ml groups resulted in lower values than those seen in the CK group, whereas those of the 10^6^ CFU/ml group were higher than the control values. The lesion diameter of the 10^7^ CFU/ml group was the lowest, that is, II < III <CK < I (*p* < .05). Regarding the DI, the 10^6^ and 10^7^ CFU/ml groups had lower values than the CK group, while there was an increasing trend in the 10^8^ CFU/ml group (Figure 3b), that is, II < I < CK < III (*p *< .05). The DI of group B (10^7^ CFU/ml) was 36%, which was 15% lower than that of the CK group. From the results, 10^7^ CFU/ml of NCPSJ7 was concluded to have a better inhibitory effect against gray mold on the grapes.

### 3.2 Effects of NCPSJ7 on resistance‐related enzymes in infected Red Globe grapes

**Figure 4:**
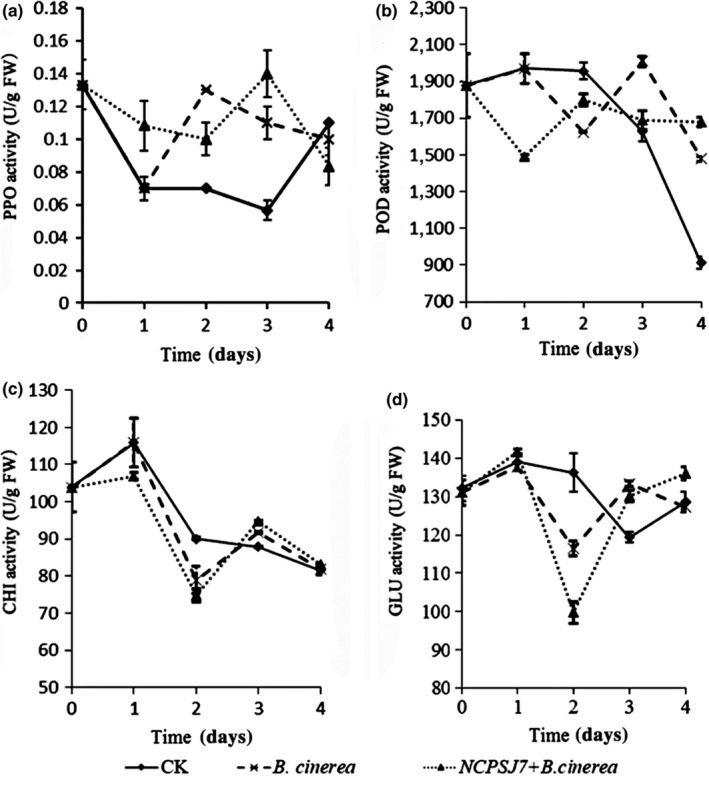
The effects of 1 × 10^7^ CFU/ml NCPSJ7 on resistance‐related enzymes in treated red globe grapes. (a) Polyphenol oxidase (PPO); (b) peroxidase (POD); (c) chitinase (CHI); and (d) β‐1,3 glucanase (GLU). Values are means ± standard errors (*n* = 3)

The PPO enzyme activity in plant tissues was significantly increased when the plant was damaged or susceptible to infection. From Figure 4a, it can be seen that the PPO activity was higher in the *B. cinerea* group and NCPSJ7 + *B. cinerea* group than in the control group between days 0 and 3. With the extension of storage time, grape lesions began to appear on the 3rd day and the PPO enzyme activity in the NCPSJ7 + *B. cinerea* group was higher than that in the *B. cinerea* group. This indicated that NCPSJ7 could enhance the enzymatic activity of PPO and effectively resist the invasion of pathogens and improve the resistance of the fruits to pathogen infection. On the 4th day, the quality of the fruit had decreased owing to the continuous propagation of *B. cinerea*. By the end of the storage period, the enzyme activities in the treated groups had decreased to values below those in the control group.

Peroxidase is a cell membrane‐protecting enzyme that eliminates free radicals in plants (Cheruth et al., 2009). As seen in Figure 4b, the POD enzyme activity of the CK group decreased gradually throughout the storage period, whereas that in group NCPSJ7 + *B. cinerea* was more stable within a certain period, although it was slightly lower than the CK group level before the 3rd day. However, on the 4th day, the trend in POD activity was NCPSJ7 + *B. cinerea* group > *B. cinerea* group > CK group, indicating that NCPSJ7 treatment can improve the activity of this enzyme. In the process of antagonistic bacterium‐induced grape resistance toward exogenous pathogens, PPO might confer protection in the early stages of resistance, whereas POD may be involved in the defense response in the middle and late stages.

As shown in Figure 4c, although the NCPSJ7 + *B. cinerea* treatment reduced the activity of CHI between days 0 and 2, the enzyme activity increased on the 3rd day. The trend was higher than that of the CK and *B. cinerea* groups and continued until the end of the experiment. This also indicated that the antagonistic bacterium *B. amyloliquefaciens* NCPSJ7 could induce strong resistance in the middle and late stages of fruit infection.

In Figure 4d, the GLU activity in the *B. cinerea* and NCPSJ7 + *B. cinerea* groups can be seen to be lower than that in the control group between days 0 and 2. However, when the bacterial lesion was observed on the 3rd day, GLU had increased rapidly in both groups until it was higher than that in the control group, indicating that the pathogen and NCPSJ7 can induce fruit resistance. On the 4th day, the activity of GLU induced by NCPSJ7 (136.04 U/g FW) was higher than that in the CK group (128.58 U/g FW) and was stronger than that in the group inoculated with the pathogen alone (127.18 U/g FW). Therefore, we concluded that the antagonistic bacterium NCPSJ7 can induce fruit resistance toward the pathogen.

### 3.3 Effects of NCPSJ7 on the antioxidative capacity in infected Red Globe grapes

**Figure 5:**
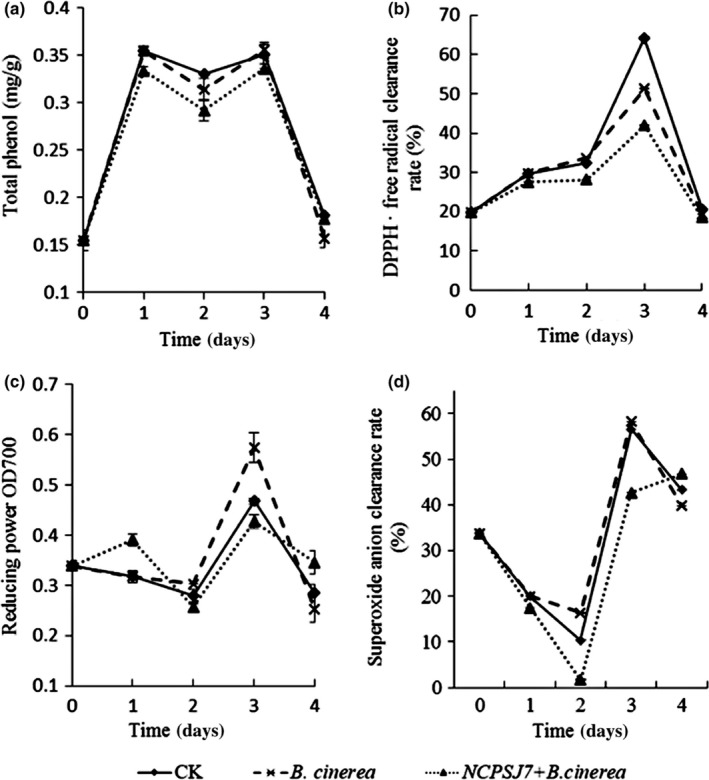
The effects of 1 × 10^7^ CFU/ml NCPSJ7 on antioxidant capacities in treated red globe grapes. (a) Total phenol; (b) DPPH free radical clearance rate; (c) reducing power; and (d) superoxide anion clearance rate. Values are means ± standard errors (*n* = 3)

Figure 5a demonstrates that all treatments showed an “M” curve trend, with two peaks appearing on days 1 and 3, respectively, during the entire storage period. The peak on the 1st day was probably due to the fruit body itself producing total phenols against external damage. The peak at day 3 was likely due to damage, along with *B. cinerea*, leading to more phenolic production, which is consistent with the data for the 3rd day (i.e., *B. cinerea* > CK>NCPSJ7 + *B. cinerea*). During the later stages of storage, the TPC in the *B. cinerea* and NCPSJ7 + *B. cinerea* groups was, respectively, lower than that in the CK group, indicating that the pathogen invasion did not lead to the increase in total phenols, and NCPSJ7 did not increase the TPC.

The DPPH free radical clearance rate is one indicator of antioxidative capacity. As shown in Figure 5b, throughout the entire storage period, all three treatments conferred a rise and then rapid fall in the free radical scavenging rate, with the antagonistic bacterium‐treated group having the lowest rates. This indicated that the presence of NCPSJ7 combined with *B. cinerea* had reduced the DPPH free radical scavenging capacity of the grapes.

As shown in Figure 5c,d, the trends of the reducing power and superoxide anion clearance rate were similar. The trend on the 3rd day was *B. cinerea* > CK>NCPSJ7 + *B. cinerea*, indicating that the antagonistic bacterial had decreased the reducing power and the superoxide anion clearance rate. On the 4th day, however, the values for the NCPSJ7 + *B. cinerea* group were higher than those for the other two groups, especially those for superoxide anion clearance, indicating that the bacteria can delay the downtrends of reducing power and superoxide anion clearance.

## 4 DISCUSSION

Gray mold has brought huge losses to the grape industry in terms of yield and economics (Ozkan, Smilanick, & Karabulut, 2011). Biological control plays an important role in fruit and vegetable postharvest preservation following the physical and chemical control means. In this study, treatment with 10^7^ CFU/ml of NCPSJ7 significantly lowered the disease incidence of grapes relative to that in the CK group at day 4, and the DI was controlled, even on the 6th day. Generally, NCPSJ7 had a good inhibitory effect on gray mold, as it reduced the disease incidence, DI, and lesion diameter.

Induction of resistance is one of the mechanisms of biological control technology to inhibit postharvest diseases of fruits and vegetables (Wang, Wang, Jin, & Zheng, 2013). PPO and POD enzymes play a key role in the catalysis of lignin synthesis; lignin can form an interlaced network to harden the cell wall, which improves the disease resistance. In our study, NCPSJ7 improved the activity of PPO, effectively conveying resistance against the invasion of pathogens during the early storage period. This result is consistent with findings of Fan, Li, and Shi (2016). In addition, POD may be involved in the defense response during the middle and late storage periods. POD and PPO showed strong activities in the different storage periods, demonstrating a synergistic effect. However, the results are inconsistent with those obtained by Qin, Tian, Liu, and Xu (2002), in which *Pichia membranefaciens* and *Rhizopus stolonifer* did not induce the POD enzyme activity in peaches. Therefore, the ability of antagonistic bacteria to induce POD enzyme activity and improve plant disease resistance depends on the type of antagonistic bacteria and pathogens present in the plant.

Chitinase and GLU, as disease‐related proteins, are considered key hydrolytic enzymes for hydrolyzing the fungal cell wall, and they play an important role in biological control (Arora, Khare, & Verma, 2008; Kowsari, Motallebi, & Zamani, 2014). Hydrolytic enzymes secreted by antagonistic bacteria usually use chitin or the pathogen cell wall as a carbon source, hydrolyze the mycelia of pathogens, and strengthen the antagonistic effects (Viswanathan, Sundar, & Premkumari, 2003). During the storage of grapes, the activity of CHI enzyme in the NCPSJ7 + *B. cinerea* group was not consistently higher than that in the CK and *B. cinereal* groups, but was strongly induced when lesions were present. The lower disease incidence in the NCPSJ7 + *B. cinerea* group proved the induction effect of NCPSJ7. The activity of GLU was consistent with that of CHI, where the similarity in the trends of these two enzymes demonstrated the bacterial synergistic effect. This finding is consistent with the findings of Zuo, Yan, Yang, and Liu (2008).

Antioxidant capacity is important for indicating the quality of the fruit (Wang et al., 2017). The antagonistic bacterium NCPSJ7 initially reduced the TPC, DPPH free radical scavenging rate, reducing power, and superoxide anion clearance rate in the early stage of lesions; however, it eventually increased the reducing power and DPPH radical scavenging rate during the course of infection. Nonetheless, it was still lower than the CK and *B. cinerea* groups. This means that NCPSJ7 caused a reduction in fruit quality. In this experiment, the method of stabbing the grapes to inoculate the antagonistic bacterium damaged the fruits. Therefore, in order to minimize the adverse effects on fruit quality, attempts should be made to avoid their mechanical injury in actual field applications by inoculating the antagonistic bacteria through other means, such as smearing and spraying. However, further studies are warranted to elucidate the effects of NCPSJ7 alone without *B. cinerea* inoculation on the enzyme activity and antioxidant capacity of grapes.

Although inducing the resistance of host enzymes was a key factor in successful control of fungal disease in fruit, lipopeptides and volatile compounds produced by *B. amyloliquefaciens* might be other possible effective biocontrol mechanisms. Arrebola, Jacobs, and Korsten (2010) reported iturin A as a lipopeptide that showed a strong inhibitory activity against seven postharvest fungi in citrus. Moreover, 3‐hydroxy‐2‐butanone, a major ketone compound, was developed by *B. amyloliquefaciens* PPCB004 to control the disease caused by *Penicillium crustosum* (Arrebola, Sivakumar, et al., 2010)*.* Thus, future experiments should investigate the relevant mechanisms of *B. amyloliquefaciens* NCPSJ7 against the fungi in infected fruit and vegetables.

## 5 CONCLUSION

We have established that 10^7^ CFU/ml of *B. amyloliquefaciens* NCPSJ7 was effective in inducing resistance against gray mold in Red Globe grapes and enhancing the activities of PPO, POD, CHI, and GLU enzymes during different storage periods. The NCPSJ7 + *B. cinerea* group improved the oxidative resistance, which was observed as an escalating trend in the total phenolic content, DPPH free radical clearance rate, reducing power, and superoxide anion clearance rate after lesion presentation, compared with *B. cinerea* alone and the control. However, although NCPSJ7 showed an inhibitory activity on gray mold, part of the antioxidant capacity decreased in grapes.

## CONFLICT OF INTEREST

The authors declare that there are no conflicts of interest.

## ETHICAL APPROVAL

This study does not involve any human or animal testing.
